# American Views on the Treatment of Appendicitis

**Published:** 1902-07-05

**Authors:** 


### AMERICAN VIEWS ON THE TREATMENT OF APPENDICITIS.

It is not without interest to note that at the annual meeting of the American Medical Association which has just been held at Saratoga Springs, the treatment of appendicitis was fully discussed, and that the most positive statements were made by many of the speakers as to the necessity for early, nay for immediate, operation. Dr. John B. Deaver and Dr. Geo. G. Ross presented a paper giving a critical review of a series of 426 cases. Out of the total number no fewer than 279 had been acute cases, and in these Dr. Deaver had a mortality of 15 per cent. The whole trend of this paper was in favour of early operation. The ice bag was held to be as bad as opium, in that it masked valuable symptoms and did no good. The uncertainty which hangs over every case of appendicitis would, it was maintained, no longer exist if every case were operated on the moment it was seen. The authors took strenuous exception to the general belief that amelioration of symptoms contraindicates operation, for all the miserable sequelæ of the disease could be avoided, they said, by early operation.

Dr. Parker Syms of New York held that in a series of fatal cases which he related, death had in every case been due to delay. There was, he said, no patient upon whom one might not at one time have operated successfully. Dr. Ernest La Place, of Philadelphia, said that an axiom with which everyone should be familiar was that at no time can any human being be sure of the extent of the disease, except after operation, and that, therefore, every case ought to be operated on at the earliest possible moment. Dr. Weir, of New York, agreed with Dr. Deaver that every case seen must be operated on at once. Dr. Murphy, of Chicago, said that the problem of when to operate was rapidly nearing solution. Practically all the members of the section were agreed that it must be within 36 hours. When the profession became educated to this the mortality would be reduced to less than one half per cent.

If we may be allowed to make a comment on what may for the present be termed the American view, which undoubtedly is drifting more and more towards immediate operation, we would suggest that in a disease which shows such an infinite variety both in the nature and degree of the pathological changes existing, these attempts to ignore all differences in regard to treatment, and to bring down the whole business to one monotonous dead level of operation, are the reverse of scientific. They appear to be the outcome of a sort of diagnostic despair, a hopelessness of ever being able to arrive at a satisfactory decision as to what cases may safely be left and what cases must of a certainty be operated upon; and notwithstanding all that is being said against what we suppose we may still call the English view, and especially against the cautious and judicious one taken by Mr. Treves, we cannot but feel that this view, which is based on the sound principle that different conditions call for different treatment, is the proper and the scientific one to accept in regard to this complicated question.

